# Idiopathic pediatric retinal artery occlusion

**DOI:** 10.4103/0301-4738.60091

**Published:** 2010

**Authors:** George J Manayath, Parag K Shah, V Narendran, Rodney J Morris

**Affiliations:** Department of Retina, Aravind Eye Hospital and Postgraduate Institute of Ophthalmology, Coimbatore, Tamil Nadu, India

**Keywords:** Retinal artery occlusion, pediatric, treatment

## Abstract

We report a case of branch retinal artery occlusion (BRAO) in a healthy young girl. An eight-year-old girl presented with sudden loss of vision in her left eye. She had a pale retina with macular edema consistent with extensive BRAO. A thorough workup was performed to determine any etiologic factor. All test results were within normal limits. Her visual acuity improved from finger counting to 20/40 over two weeks, on immediate treatment with intravenous steroids (methyl prednisolone). This case suggests that BRAO can occur in healthy children without any detectable systemic or ocular disorders and a dramatic improvement may be achieved with prompt treatment with intravenous steroids.

Retinal artery occlusion is an extremely rare diagnosis in the pediatric population and most of these patients have some detectable etiologic disorder.[1] Hypercoagulable states and emboli are the most frequent etiologies.[2] The aim of this report is to present the case of a child with an idiopathic branch retinal artery occlusion (BRAO), who responded well to intravenous steroids.

## Case Report

**Figure 1:**
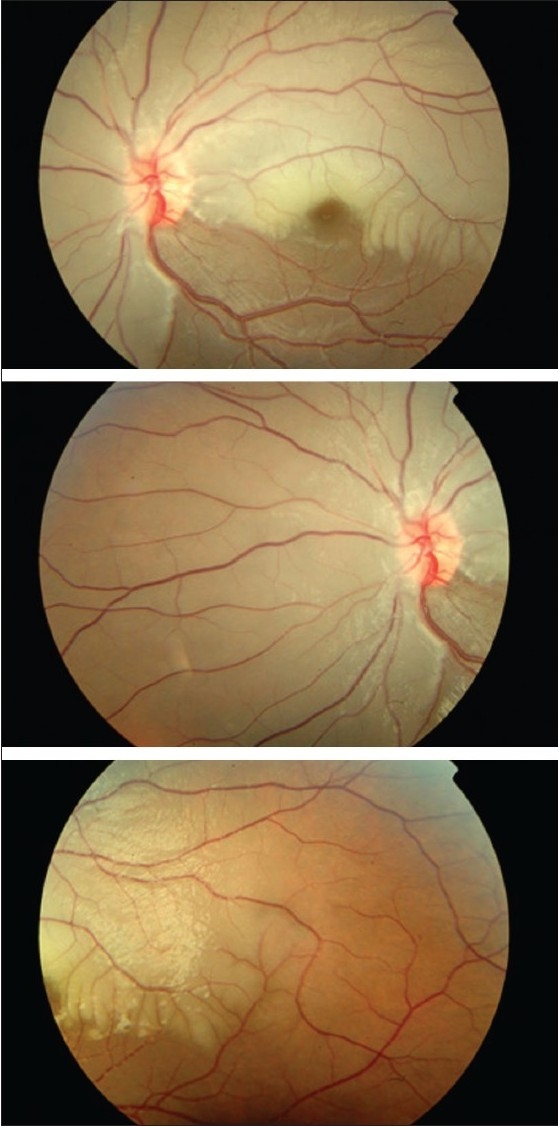
Left eye fundus photograph at presentation showing pallid retinal edema of posterior pole sparing infero-temporal quadrant, arteriolar attenuation and segmentation of blood column in arterioles, suggestive of extensive branch retinal artery occlusion

An eight-year-old girl presented to us with less than 24 h of painless decreased vision in her left eye (LE). She had no recent history of infections, trauma, systemic malignancy or other systemic complaints. Ocular examination revealed a visual acuity of counting fingers close to face in LE and 20/20 in right eye (RE). Anterior segment showed relative afferent pupillary defect in LE. There was no evidence of vitritis. Dilated fundus examination of LE revealed pallid retinal edema involving the posterior pole, all around the optic disc and macula, but sparing the infero-temporal quadrant suggestive of an extensive BRAO [Fig. 1]. There was arteriolar attenuation and segmentation of blood column within the arterioles. Induced central retinal artery pulsations at the disc were present suggesting a partial occlusion. The RE was normal.

Etiological workup by way of cardiovascular evaluation including echocardiography and carotid Doppler study was within normal limits. Similarly, blood investigations including complete blood count with Erythrocyte sedimentation rate ESR, peripheral blood smear, platelet count, Prothrombin/Activated Partial Thromboplastin time, serum lipids, autoimmune markers (Antinuclear, Anticardiolipin (Antiphospholipid), Antineutrophilic cytoplasmic (ANCA) antibodies), hypercoagulability testing (fibrinogen, Antithrombin III, Proteins C and S, Factor V Leiden assay) and serum homocysteine were found to be within normal limits. Infectious disease workup revealed a negative Mantoux test, *Treponema Pallidum* Hemagglutination (TPHA) and Toxoplasma titers.

**Figure 2:**
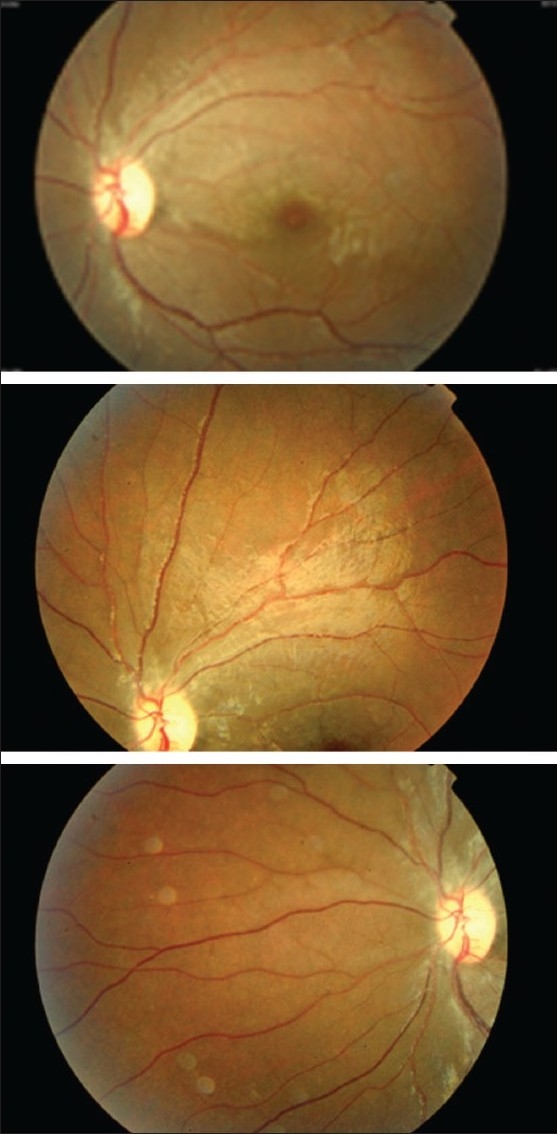
Left eye fundus photograph showing almost complete resolution of pallid retinal edema, two weeks following the treatment

On the same day of presentation, ocular massage in supine position followed by anterior chamber paracentesis under aseptic conditions and short general anesthesia was performed and she was empirically started on Aspirin (5 mg/kg/day) and intravenous methyl prednisolone (20 mg/kg/day). After three days, she was started on a tapering dose of oral steroids. Visual acuity improved to 20/200 on the third day and to 20/40 by two weeks. The retinal pallid edema had resolved remarkably [Fig. 2]. Follow-up three months later revealed an almost normal fundus and the vision was stable at 20/30.

## Discussion

The average age of patients with retinal artery occlusion is 58.5 years.[1] The incidence of retinal arterial obstruction in patients under the age of 30 years has been estimated at less than 1 in 50,000 outpatients.[1] Frequent etiologies include hypercoagulable states, emboli from cardiac valvular disease, and known risk factors such as smoking, oral contraception, and vasospasm as in migraine histories.[1,2] The youngest reported arterial obstruction is that of a 40-day-old female neonate with shock and diffuse intravascular coagulation (DIC), who developed bilateral ophthalmic artery occlusion.[3]

Retinal artery occlusion is associated with a poor visual prognosis, and aggressive management with ocular massage, anterior chamber paracentesis, and carbogen therapy does not appear to improve the outcome.[4] Retinal arterial occlusions in the young typically occur by somewhat different mechanisms than do arterial occlusions in adults. In our case no underlying etiology was found despite extensive investigation, and the presence of extensive BRAO in the absence of a cardio-vascular source of emboli prompted us to think in terms of an inflammatory etiology leading to vasculitis and occlusion, particularly considering the age group of the patient. Pediatric retinal artery occlusions are reported to have poor visual outcome, till date.[5–7] Therefore, after discussions with the pediatrician and the child's parents, we started a course of intravenous steroids.

To date, two reports exist of idiopathic central retinal arterial occlusions (CRAO) in the pediatric age group.[5,6] The first report describes the case of a six-year-old boy who was diagnosed CRAO with disc swelling, approximately 50 h since the onset of CRAO, as the condition was initially misdiagnosed as neuro-retinitis due to the age group of the patient.[5] The second describes the case of an eight-year-old boy with CRAO and disc edema and no definite etiology, but a four-day history of flu-like symptoms led the authors to attribute the pathology to a post-viral vasculitis.[6] Both the cases were extensively investigated and both the cases had poor visual outcome. There is also a report of a case of BRAO, in an 11-year old girl with permanent unilateral visual field loss in the right eye and no underlying cause found after extensive investigations.[7]

In this instance, we report a case successfully treated by intravenous steroids given under the presumed diagnosis of vasculitis as the cause of retinal artery occlusion. Though retinal arterial obstruction is rare in children, our case highlights the importance of thorough investigation and prompt and appropriate treatment.
